# Caregivers' Cannabis Use: Does Burden Lead to Blunts?

**DOI:** 10.1093/geroni/igab046.1966

**Published:** 2021-12-17

**Authors:** Kanika Arora

**Affiliations:** University of Iowa, Iowa city, Iowa, United States

## Abstract

Research on risky health behaviors among caregivers is limited. In this paper, we examine the association between informal caregiving and marijuana use and whether this association varies by age. Using data from Behavioral Risk Factor Surveillance System (2016-2019), a multivariable logistic regression model compared marijuana use in “caregivers” and “expectant caregivers.” We stratified the analyses by age and also assessed the association between caregiving intensity and marijuana use. Among younger individuals (18-49 years), informal caregiving was associated with higher odds of marijuana use. In this group, higher prevalence of marijuana use was positively associated with care intensity. There was no detectable association between caregiving and marijuana use among older individuals (50 years or older). Health behaviors among caregivers differ by age. Combined exposure to informal caregiving and marijuana in young adulthood may lead to adverse long-term health consequences. Immediate effects of marijuana use may negatively influence care recipient outcomes.

